# The neurological and ophthalmological manifestations of *SPG4*-related hereditary spastic paraplegia

**DOI:** 10.1007/s00415-012-6780-3

**Published:** 2012-12-13

**Authors:** Grant Guthrie, Gerald Pfeffer, Maura Bailie, Karen Bradshaw, Andrew C. Browning, Rita Horvath, Patrick F. Chinnery, Patrick Yu-Wai-Man

**Affiliations:** 1 Department of Ophthalmology, Royal Victoria Infirmary, Newcastle upon Tyne, UK; 2 Department of Neurology, Royal Victoria Infirmary, Newcastle upon Tyne, UK; 3 Wellcome Trust Centre for Mitochondrial Research, Institute of Genetic Medicine, Newcastle University, Newcastle upon Tyne, NE1 3BZ UK; 4 Department of Medical Physics, Royal Victoria Infirmary, Newcastle upon Tyne, UK

Dear Sirs,

The hereditary spastic paraplegias (HSPs) are a genetically heterogeneous group of disorders characterised by progressive corticospinal tract degeneration and the development of lower limb spasticity [1, 2]. Autosomal-dominant HSP is the most commonly inherited form of the disease and in this group, *SPG4* mutations account for ~40 % of cases [1]. The *SPG4* gene codes for spastin, a critical neuronal protein that maintains organelle axonal transport by severing and rearranging the microtubule network [3, 4]. Dysfunctional mutant proteins or insufficient quantities of the wild-type protein inhibit this dynamic shuttling process resulting in axonal swelling and progressive retrograde degeneration that preferentially affects the long corticospinal axons [3, 4].

There is mounting evidence that the microtubule and mitochondrial networks are intrinsically linked at the cellular level [5]. This intriguing association has recently been highlighted by the clinical observation that autosomal-dominant optic atrophy (DOA)—a classical mitochondrial optic neuropathy caused by pathogenic *OPA1* mutations—can result in complicated neurological phenotypes (DOA+) with features indistinguishable from HSP [6]. Furthermore, subclinical corticospinal tract dysfunction also seems to be a prevalent feature among *OPA1* mutation carriers presenting with isolated visual failure, suggesting a wider disease spectrum than originally considered [7]. Interestingly, these overlapping genotype-phenotype manifestations have been reported previously in families with a rarer, autosomal-recessive form of HSP caused by pathogenic *SPG7* mutations, in which bilateral optic atrophy was a prominent feature segregating with spastic paraplegia [8–10]. Given the emerging disease mechanisms linking corticospinal tract dysfunction with optic nerve degeneration, the aim of this study was to determine the neurological and ophthalmological manifestations of *SPG4*-related HSP, looking specifically for evidence of clinical or subclinical optic neuropathy among affected patients. The overall neurological disability, including cognitive function, was also evaluated to provide a comprehensive assessment of the burden of disease in this group of patients.

A comprehensive neurological (GP, RH, PFC) and ophthalmological (PYWM) assessment (Supplementary Method) was carried out on ten white patients from the North of England harbouring confirmed pathogenic *SPG4* mutations (Table 1). A broad spectrum of neurological disability was observed among affected patients with scores ranging from one to nine on the modified EDSS scale (Table 2). Importantly, four patients had abnormal MOCA scores of less than 26 points. Seven patients performed poorly on the memory component of the MOCA test protocol and one patient had abnormal visuospatial/executive performance. The association between *SPG4* mutations and progressive cognitive decline remains controversial [11–13] and our study of a well-characterised patient cohort provides further evidence favouring a true causal link. Two of the patients with abnormal MOCA scores were younger than the age of 30 years, clearly highlighting the need for clinical vigilance to detect early signs of cognitive impairment and to provide adequate level of support, especially to carers.

**Table 1:** Molecular genetic and ophthalmological features of the *SPG4* patient cohort

Patient	Sex	Age (years)	SPG4 mutation		BCVA RE-LE	Optic discs/OCT measurements	Eye movements	Visual electrophysiology
			Exon	cDNA change/consequence				
1	F	31	5	c.743C>G/p.S245X	20/20-20/20	Normal/no RNFL thinning	Normal	Normal
2	M	53	5	c.743C>G/p.S245X	20/20-20/20	Normal/no RNFL thinning	Horizontal SWJ/saccadic pursuit	Normal
3	F	50	6	c.937delG/p.D313fsX1	20/20-20/20	Normal/no RNFL thinning	Normal	Normal
4	F	55	4–17	del exon 4-17/large-scale deletion	20/20-20/20	Normal/no RNFL thinning	Normal	Normal
5	F	29	10	c.1253_1255delAAG/p.E418fsX198	20/20-20/20	Normal/no RNFL thinning	Horizontal SWJ/saccadic pursuit	Normal
6	F	25	11	c.1442_1443insA/p.V482fsX5	20/20-20/20	Normal/no RNFL thinning	Normal	Normal
7	F	55	11	c.1442_1443insA/p.V482fsX5	20/20-20/20	Normal/no RNFL thinning	Horizontal SWJ/saccadic pursuit	Normal
8	F	49	11	c.1414G>A/p.V472I	20/20-20/20	Normal/no RNFL thinning	Normal	Normal
9	F	72	11	c.1384A>G/p.K462E	20/60-20/30	Normal/no RNFL thinning	Normal	Normal
10	M	65	11	c.1081C>A; c.1082T>A/p.L361N	20/20-20/20	Normal/no RNFL thinning	Normal	Normal

*BCVA* best-corrected visual acuities, *cDNA* complementary DNA, *LE* left eye, *OCT* optical coherence tomography, *RE* right eye, *RNFL* retinal nerve fibre layer, *SWJ* square wave jerks

**Table 2:** Neurological and cognitive features of the *SPG4* patient cohort

Patient	Cognitive assessment (MOCA)		Motor examination											Vibration sense	Other findings	Disability measurements	
			Modified Ashworth spasticity score		Deep tendon reflexes						Power (MRC scale)		Coordination				
	Score	Comments	L/R E	L/R K	L/R BR	L/R B	L/R T	L/R P	L/R A	PR	UL ex/fl	LL fl/ex				10 m walk	Modified EDSS
1	27/30	3 points from memory	0, 0	3, 3	3, 3	3, 3	3, 3	4, 4	2, 2	Ex	5, 5	4, 5	AT	Ankles		19.1 s	6.5
2	27/30	4 points from memory	0, 0	1, 1	2, 2	2, 2	2, 2	3, 3	2, 2	Fl	5, 5	5, 5	N	N		8.2 s	1
3	27/30	2 points each from visuospatial/executive and attention	0, 0	1, 1	2, 2	2, 2	2, 2	3, 3	2, 2	Ex	5, 5	4, 4	N	Knees		10.1 s	2
4	27/30	3 points from memory	0, 0	2, 2	3, 3	3, 3	2, 2	3, 3	2, 2	Ex	5, 5	4, 3–4	N	Knees	Marked LL oedema	WC	7
5	23/30	3 points from memory	3, 3	4, 4	3, 3	3, 3	3, 3	3, 3	4, 4	Ex	4, 4	0, 0	N	Knees	Spastic dysarthria	WC	9
6	25/30	3 points from memory	0, 0	1, 1	2, 2	2, 2	2, 2	3, 3	3, 3	Fl	5, 5	5, 5	N	N		8.9 s	1
7	25/30	5 points from memory	0, 0	3, 3	2, 2	3, 3	3, 3	4, 4	3, 3	Ex	5, 5	4, 5	N	Ankles		13.4 s	4
8	29/30		0, 0	3, 3	2, 2	2, 2	2, 2	4, 4	4, 4	Ex	5, 5	4, 4	N	N		11.3 s	2
10	25/30	4 points from memory	1, 1	2, 2	2, 2	2, 2	2, 2	3, 3	3, 3	Ex	5, 5	4, 4	N	Ankles	Two canes needed for walking	36.6 s	6.5

Patient nine was not available for this portion of the assessment. A total MOCA score of 26 or higher is considered normal. For subjects with 12 years of total education or less, an additional point is added. MOCA scores lower than 26 are suggestive of cognitive impairment [14]. Vibration sense has been reported as the lowest normal testing location. Published normative range for the 10 m walk test protocol: mean 6.7 s, 95 % confidence interval 5.6–7.9 s [15]

*A* Achilles, *AT* action tremor, *B* biceps, *BR* brachioradialis, *E* elbow, *EDSS* Expanded Disability Status Scale, *Ex* extensor, *Fl* flexor, *K* knee, *L* left, *LL* lower limbs, *m* metres, *MOCA* Montreal Cognitive Assessment Scale, *MRC* Medical Research Council, *N* normal, *PR* plantar responses, *R* right, *s* seconds, *T* triceps, *UL* upper limbs, *WC* wheelchair-bound

Except for one patient who had bilateral nuclear sclerotic cataracts, all patients had best-corrected visual acuities of 20/20 bilaterally (Table 1). The ophthalmological examination was normal with full colour discrimination and no detectable optic disc or retinal abnormalities. Visual fields, RNFL thickness measurements and visual electrophysiology were within the normal range for the entire HSP patient cohort. Three patients had abnormal eye movements with horizontal square wave jerks and saccadic smooth pursuits. No significant ptosis or limitation of eye movements was noted on orthoptic assessment. Based on our comprehensive clinical and electrophysiological evaluation, visual loss secondary to optic nerve or retinal degeneration is unlikely to be a major phenotypic manifestation of *SPG4*-related disease. Affected patients and at-risk family members can therefore be reassured that unlike other genetically-determined forms of HSP [6–10], *SPG4* mutations are not associated with the development of significant ophthalmological complications, in particular visual failure.

## Electronic supplementary material

Below is the link to the electronic supplementary material.

Supplementary material 1 (DOC 80 kb)
