# Supplementary material for: The neurological and ophthalmological manifestations of SPG4-related hereditary spastic paraplegia
Source: J Neurol. 2012 Dec 13;260(3):906–9. doi: 10.1007/s00415-012-6780-3 (PMC3590400; doi:10.1007/s00415-012-6780-3)
Supplement: Supplementary file 1 — Supplementary material 1 (DOC 80 kb) [file 415_2012_6780_MOESM1_ESM.doc]

## Supplementary Method

### Neurological Assessment

Spasticity was graded at the elbows and at the knees using the modified Ashworth spasticity scale (**Supplementary Table 1**) [1]. Cognitive performance was evaluated using the Montreal Cognitive Assessment (MOCA) protocol [2]. Gait was assessed based on the average time taken to complete three trials of a 10-metre walk [3]. The degree of neurological disability was quantified using the Expanded Disability Status Scale (EDSS), which was modified to include only motor and ambulatory components (**Supplementary Table 2**) [4].

### Ophthalmological Assessment

Dilated slit-lamp biomicroscopy was carried out on all patients. Colour discrimination was assessed with the Ishihara pseudoisochromatic plates. Formal perimetry was carried out with a Humphrey^TM^ visual field analyzer operating the 24:2 Swedish Interactive Threshold Algorithm (SITA) standard testing strategy. Macular and peripapillary retinal nerve fibre layer (RNFL) thickness was measured with the acquisition protocols on a time domain Stratus OCT^TM^ (Carl Zeiss Meditec) [5, 6]. Visual electrophysiological testing was performed by an experienced operator (KB) in accordance with the minimum standards of the International Society for Clinical Electrophysiology of Vision [7]. The test protocol included pattern reversal visual evoked potentials, multifocal, full-field and pattern electroretinograms recorded with gold foil recording electrodes. This study had the relevant institutional ethical approval and complied with the Declaration of Helsinki.

Supplementary Table 1. Scoring system for the modified Ashworth spasticity scale

| **Score** | **Description** |
| --- | --- |
| 0 | Normal motor tone |
| 1 | Slight spasticity at end of range of motion |
| 1+ | Spasticity in less than half of range of motion |
| 2 | Spasticity through most of range of motion but still easily moved |
| 3 | Spasticity through full range of motion, passive movement difficult |
| 4 | Rigidity in flexion or extension |

Adapted from Bohannon and Smith, 1987 [1].

Supplementary Table 2. Scoring system for the modified Expanded Disability Status Scale (EDSS)

| **Score** | **Description** |
| --- | --- |
| 0 | Normal exam |
| 1 | Minimal signs, no disability |
| 2 | Mild disability (no assistance), fully ambulatory |
| 3 | Moderate disability (assists needed), fully ambulatory |
| 4 | Moderate disability and ambulatory up to 500m unassisted/without rest, 12 hour days self-sufficient |
| 4.5 | Moderate disability and ambulatory up to 300m unassisted/without rest, some limitation of self-sufficiency |
| 5 | Ambulatory without aid up to 200m, provisions required for full work day |
| 5.5 | Ambulatory without aid up to 100m, cannot work full day |
| 6 | Ambulation with cane required for 100m |
| 6.5 | Bilateral canes required for 20m |
| 7 | Ambulation 5m with aids |
| 7.5 | Wheelchair-restricted, aids with transfers, few steps only |
| 8 | Bed-wheelchair restricted, still good use of arms, retains much self-care |
| 8.5 | Bed-wheelchair restricted, retains some self-care |
| 9 | Bed restricted but can communicate/eat |
| 9.5 | Bed restricted and cannot communicate/eat effectively |

Adapted from Kurtzke, 1983 [4].

## Supplementary References

1. Bohannon RW, Smith MB (1987) Interrater Reliability of a Modified Ashworth Scale of Muscle Spasticity. Phys Ther 67:206-207.

2. Nasreddine ZS, Phillips NA, Bedirian V *et al* (2005) The montreal cognitive assessment, MoCA: A brief screening tool for mild cognitive impairment. J Am Geriatr Soc 53:695-699.

3. Watson M (2002) Refining the Ten-metre Walking Test for Use with Neurologically Impaired People. Physiotherapy 88:386-397.

4. Kurtzke JF (1983) Rating Neurologic Impairment in Multiple-Sclerosis - an Expanded Disability Status Scale (EDSS). Neurology 33:1444-1452.

5. Milea D, Sander B, Wegener M *et al* (2009) Axonal loss occurs early in dominant optic atrophy. Acta Ophthalmol 88:342-346.

6. Yu-Wai-Man P, Bailie M, Atawan A, Chinnery PF, Griffiths PG (2011) Pattern of retinal ganglion cell loss in dominant optic atrophy due to *OPA1* mutations. Eye 25:597-601.

7. Holder GE, Brigell MG, Hawlina M, Meigen T, Vaegan, Bach M (2007) ISCEV standard for clinical pattern electroretinography--2007 update. Doc Ophthalmol 114:111-6.
